# Underrated and Underestimated – Deprivation in Dementia. A Case Report

**DOI:** 10.1192/j.eurpsy.2024.1321

**Published:** 2024-08-27

**Authors:** S. C. Steininger, R. Frizi-Roser, S. Schneider, T.-V. Hoang, T. Zetzsche

**Affiliations:** Alterspsychiatrie, Clienia Schlössli, Oetwil am See, Switzerland

## Abstract

**Introduction:**

Deprivation is widely known in children and adolescents and means a lack of social, emotional, or sensory stimuli, due to disabilities such as deafness, but also social isolation and reduced parental care. It may cause developmental disorders such as impaired language, motoric and social development. Little is known of the impact of social deprivation in demented patients.Stimulus shielding, which is a widespread option for psychiatric symptoms of dementia such as agitation, vocalization and aggressive behavior may – if frequently used- have similar effects on demented patients.

**Objectives:**

We report the case of a 71-year-old patient with dementia caused by PSP (Progressive Supranuclear Palsy), who was in inpatient treatment due to continuous undirected vocalizations. She presented with inability to walk, dysarthria, aphasia, and hearing difficulties beside major mnestic impairment. In a prior hospitalization and in her residency, she was frequently isolated from other patients due to loud screaming and vocalizations in terms of stimulus shielding by suspected overstimulation. In order to that, for four months, she developed progressive difficulties to speak, hear, understand, as well as gait disorders. In addition, the vocalizations increased.

**Methods:**

We rated the symptoms due to deprivation, triggered by lack of mobilization, social experiences, visual, tactile and acoustic stimuli following a vicious circle of anxiety, vocalizations and recurrent isolations. Therefore, a multimodal therapy assessment was implemented, including daily physical therapy, mobilization, basal stimulation, social reintegration and basal conversation training.

**Results:**

After a few days of high intensity treatment, speech reappeared in form of one- word sentences and proceeded to the ability to have short conversations. Mobility increased, starting from severe gait disorder, including the use of a wheelchair and emerged to the ability of walking up to 50 metres. Additionally, the undirected vocalizations improved and were reduced. In addition, hearing ability improved during the four-week treatment.

**Conclusions:**

This case highlights the impact of deprivation in demented patients. Especially it shows that these symptoms can be reversible under a high intensity multimodal and multi- professional treatment within a few weeks. Therefore, stimulus shielding, should be carefully evaluated in order to prevent deprivation – and thus deterioration of the symptoms – in demented patients.

**Disclosure of Interest:**

None Declared

